# Data on the number and frequency of scientific literature citations for established medulloblastoma cell lines

**DOI:** 10.1016/j.dib.2016.10.004

**Published:** 2016-10-13

**Authors:** D.P. Ivanov, D.A. Walker, B. Coyle, A.M. Grabowska

**Affiliations:** a Cancer Biology, Division of Cancer and Stem cells, University of Nottingham, UK; b Children׳s brain tumour research centre, University of Nottingham, UK

**Keywords:** WNT, SHH, Group 3, Group 4, Molecular subgroups, Preclinical models

## Abstract

This article collates information about the number of scientific articles mentioning each of the established medulloblastoma cell lines, derived through a systematic search of Web of Science, Scopus and Google Scholar in 2016. The data for each cell line have been presented as raw number of citations, percentage share of the total citations for each search engine and as an average percentage between the three search engines. In order to correct for the time since each cell line has been in use, the raw citation data have also been divided by the number of years since the derivation of each cell line. This is a supporting article for a review of *in vitro* models of medulloblastoma published in “in vitro models of medulloblastoma: choosing the right tool for the job” (D.P. Ivanov, D.A. Walker, B. Coyle, A.M. Grabowska, 2016) [1].

**Specifications Table**

**Table:** 

Subject area	*Biology*
More specific subject area	*Children׳s brain tumours - Medulloblastoma*
Type of data	*Excel file*
How data was acquired	*Systematic search of Web of Science, Google Scholar and Scopus databases*
Data format	*Raw, analysed*
Experimental factors	*The name of each cell line was searched along with the term medulloblastoma*
Experimental features	*Raw data were transformed to number of citations per year since cell line derivation or publication*
Data source location	*Nottingham, UK*
Data accessibility	*Data is shared within this article*

**Value of the data**

• The data shows the relative popularity for each of the established medulloblastoma cell lines.
• The most frequently cited cell lines can be easily seen along with the number of papers where they are mentioned.
• In conjunction with the review article, the data can be used to link cell lines and medulloblastoma subtypes.
• Researchers can readily identify underrepresented medulloblastoma subtypes, such as WNT and Group 4.
• The relative merits of using Google Scholar, Web of Science and Scopus to find information about specific cell lines can be compared.

## 1 Data

Data are organised in three spreadsheets within one Excel file. The “Raw Citations” spreadsheet gives the numbers of citations for each cell line as determined by a search of Google Scholar, Web of Science and Scopus databases. It also includes the date of derivation for each cell line and the difference with the current year (2016). The second spreadsheet named “Analysis” includes a calculation of the relative percentage of the citations for each cell line compared to the total number of citations; first for each search engine, and then as a mean of the percentage results for all three search engines. The same procedure is applied to the citation data divided by the number of years since the first publication of the cell line. Cell lines are then ranked for their relative popularity both before and after correcting for the time they have been in use. The ranks have been used to create the pie charts displaying the relative frequency of scientific mentions for the 15 most popular cell lines in Figure 2 of [1]. The third spreadsheet contains a table of the four medulloblastoma subtypes along with their reported relative frequency in patients [2], [3].

## 2 Experimental design, materials and methods

Web of Science, Scopus and Google scholar databases were searched using the name of each cell line and the term medulloblastoma. The number of all citations in Google Scholar and the number of original articles (Web of Science, Scopus) mentioning the keywords were documented for each cell line. Wildcards were used to find citations encompassing the different ways of spelling the cell lines. For example “D$283 and medulloblastoma” to find all mentions of “D-283, D 283 or D283”. The advantage of Web of Science and Scopus are that only original articles can be selected, while Google Scholar includes “grey literature”, such as conference proceedings and scientific posters. Nevertheless, Google Scholar can retrieve citations documenting the use of cell lines from the body of the articles without them necessarily being mentioned in the topic, abstract or keywords.

The list of medulloblastoma cell lines was compiled from Cellosaurus, the controlled vocabulary of cell lines developed by the Swiss Institute of Bioinformatics [4], and the review by Xu et al. [5]. The relative percentage of citations for each cell line was determined separately for each database and reported as the mean percentage for three databases. The time of the first publication was used to determine the number of years the cell line has been available for, and the difference with the current year (2016) was used to normalise the citation data.

## References

[1] Ivanov D.P., Walker D.A., Coyle B., Grabowska A.M. (2016). in vitro models of medulloblastoma: choosing the right tool for the job. J. Biotechnol..

[2] Taylor M.D., Northcott P. a, Korshunov A., Remke M., Cho Y.-J., Clifford S.C. (2012). Molecular subgroups of medulloblastoma: the current consensus. Acta Neuropathol..

[3] Shih D.J.H., Northcott P. a, Remke M., Korshunov A., Ramaswamy V., Kool M. (2014). Cytogenetic prognostication within medulloblastoma subgroups. J. Clin. Oncol..

[4] Bairoch A, The Cellosaurus: a cell line knowledge resource., Swiss Inst. Bioinforma. (n.d.). 〈http://web.expasy.org/cellosaurus/〉. 10.7171/jbt.18-2902-002 PMC5945021 29805321

[5] Xu J., Margol A., Asgharzadeh S., Erdreich-Epstein A. (2015). Pediatric brain tumor cell lines. J. Cell. Biochem..

