# Supplementary material for: Data on the number and frequency of scientific literature citations for established medulloblastoma cell lines
Source: Data Brief. 2016 Oct 13;9:696–8. doi: 10.1016/j.dib.2016.10.004 (PMC5079239; doi:10.1016/j.dib.2016.10.004)
Supplement: Supplementary file 1 — Supplementary material [file mmc1.docx]

**Conflict of interests**

*Data article*

Title: *Data on the number and frequency of scientific literature citations for established medulloblastoma cell lines*

**Authors: Ivanov DP^1^, Walker DA^2^, Coyle B^2^, Grabowska AM^1^**

Affiliations: ^1^ Cancer Biology, Division of Cancer and Stem cells, University of Nottingham, UK

^2^Children’s brain tumour research centre, University of Nottingham, UK

Contact email: delyan.ivanov@nottingham.ac.uk

**All authors declare no conflicts of interest.**
